# Ultraviolet-Based Pathogen Inactivation Systems: Untangling the Molecular Targets Activated in Platelets

**DOI:** 10.3389/fmed.2018.00129

**Published:** 2018-05-07

**Authors:** Peter Schubert, Lacey Johnson, Denese C. Marks, Dana V. Devine

**Affiliations:** ^1^Canadian Blood Services, Vancouver, BC, Canada; ^2^Centre for Blood Research, University of British Columbia, Vancouver, BC, Canada; ^3^Research and Development, Australian Red Cross Blood Service, Sydney, NSW, Australia; ^4^Sydney Medical School, The University of Sydney, Sydney, NSW, Australia

**Keywords:** platelets, pathogen inactivation, transfusion, mechanisms, signaling

## Abstract

Transfusions of platelets are an important cornerstone of medicine; however, recipients may be subject to risk of adverse events associated with the potential transmission of pathogens, especially bacteria. Pathogen inactivation (PI) technologies based on ultraviolet illumination have been developed in the last decades to mitigate this risk. This review discusses studies of platelet concentrates treated with the current generation of PI technologies to assess their impact on quality, PI capacity, safety, and clinical efficacy. Improved safety seems to come with the cost of reduced platelet functionality, and hence transfusion efficacy. In order to understand these negative impacts in more detail, several molecular analyses have identified signaling pathways linked to platelet function that are altered by PI. Because some of these biochemical alterations are similar to those seen arising in the context of routine platelet storage lesion development occurring during blood bank storage, we lack a complete picture of the contribution of PI treatment to impaired platelet functionality. A model generated using data from currently available publications places the signaling protein kinase p38 as a central player regulating a variety of mechanisms triggered in platelets by PI systems.

## The Challenges of Platelet Transfusions

Platelets play an essential role in hemostasis, fibrinolysis, and vascular integrity, which are critical physiological processes to prevent and control bleeding (1–3). Platelet concentrates (PCs) are transfused to treat bleeding in thrombocytopenic, trauma, or surgery patients (4–6) as well as for prophylactic treatment of patients with hypoproliferative thrombocytopenia (7, 8). Over the last decades, development of improved therapies and the subsequent introduction of new transfusion guidelines have changed the practice of platelet transfusion (9, 10) which has, in turn, influenced the management of platelet inventories in the blood bank.

Additionally, the integrity and safety of platelet preparations could be compromised by the presence of pathogens, such as viruses, bacteria, and parasites (11). Serious complications or death due to bacterially contaminated units have been well documented, leading to several changes in the collection procedures, including stricter donor screening, improved skin disinfection methods and diversion of the first few milliliters of collected blood, and bacterial culture of PCs (12–16). However, the risk still exists, not only for undetected bacterial contamination but for the increasing number of emerging and re-emerging pathogens, particularly viruses for which screening tests may not be in place.

Finally, even with the use of pre-storage leukoreduction, the transfer of residual allogeneic donor leukocytes in PCs still occurs and can potentially cause adverse reactions in certain platelet recipients (17). All pathogen inactivation (PI) systems show inactivation capacity of these residual leukocytes (18, 19).

These challenges of platelet storage have led to the development and increasing implementation of PI technologies which are based on ultraviolet (UV) light-mediated damage of nucleic acids and subsequent inactivation of most pathogens as well as passenger white blood cells.

## A Brief Overview of Current PI Systems

Currently, three PI systems to produce pathogen-reduced PCs are commercially available, utilizing UV in the presence or absence of a photosenzitizer. These technologies are extensively reviewed in the literature (20–29); therefore, only key points necessary for the context of this review are provided.

The INTERCEPT system (Cerus Corporation, Concord, CA, USA) uses amotosalen as photosensitzer in combination with UVA light (320–400 nm). Amotosalen penetrates the cellular membrane forming non-covalent links between pyrimidine residues in DNA and RNA. UV illumination induces a photochemical reaction that transforms the preexisting link into an irreversible covalent bond, preventing DNA replication and RNA transcription. Excess amotosalen and its photoproducts need to be removed by an in-line compound absorption device (30, 31).

The MIRASOL system (Terumo BCT, Lakewood, CO, USA) uses vitamin B2 (riboflavin) as the photosensitizer and UVA/UVB light (270–360 nm) (32, 33). In the presence of riboflavin, illumination generates free oxygen radicals causing irreversible damage to guanidine nucleotide bases. Riboflavin does not need to be removed following illumination as it is a common dietary element and generally considered to be safe.

The THERAFLEX-UV Platelets system (MacoPharma, Tourcoing, France) uses UVC light in combination with strong agitation which facilitates light penetration and does not require a photosensitizer. UVC acts directly on nucleic acids to induce pyrimidine dimers to block DNA replication (34, 35).

## Pathogen-Reduced Platelet Products

Pathogen-reduced PCs can be obtained by direct treatment of platelet components using a PI system, or they can be derived by treating whole blood with the MIRASOL (36, 37) or potentially the INTERCEPT system once a current trial turns out to be successful followed by processing into the (platelet) components (Table 1).

**Table 1:** Overview of pathogen inactivation (PI) treatment options to obtain pathogen-reduced platelet products.

Product treatment	Storage solution	PI system		
		
		INTERCEPT	MIRASOL	THERAFLEX
AP/PC	Plasma	+	+	−
	PAS	+	+	+
PRPC or BC/PC	Plasma	+	+	−
	PAS	+	+	+
WB (prior to PRPC or BC/PC production)	Plasma	−	+	−
	PAS	−	+	−

*AP/PC, apheresis platelet concentrates; PRPC, platelet-rich plasma concentrate; BC/PC, buffy-coat-derived platelet concentrate; WB, whole blood; PAS, platelet additive solution*.

It is noteworthy to point out that the THERAFLEX system require PCs produced in platelet additive solution (PAS) while the MIRASOL and INTERCEPT systems can treat PCs in plasma or PAS.

## Ongoing Debate: Safety vs Efficacy of PI

More than a decade ago, the interest in PI prompted many large-scale discussions (38–40). The outcome of these deliberations included the provision of information required for implementation of PI systems such as implementation criteria, component specifications, licensing requirements, and the impact in blood product inventories, as well as clinical issues such as transfusion efficacy, risk management issues, and cost–benefit assessments. Since then, numerous studies have been conducted to provide answers to questions on product safety, clinical efficacy, and quality.

In order to assess inactivation efficacy, studies spiking pathogens relevant to blood transfusion into PCs prior to illumination have been performed (34, 41–44). All PI systems currently on the market have demonstrated effectiveness in inactivating most tested pathogens with moderate to highly effective inactivation capacities for several emerging viruses including West Nile virus (45), chikungunyah virus (46), Zika virus (47, 48), dengue virus (49), and hepatitis-E-virus (50). Additionally, a comparative study (51) revealed that HIV-1 can be similarly inactivated by MIRASOL and INTERCEPT, however, less efficient compared to other viruses due to its resistance to UV light. Furthermore, INTERCEPT demonstrated a higher inactivation capacity for bovine viral diarrhea virus and pseudorabies virus compared to MIRASOL while both technologies showed similar log reductions for hepatitis-A-virus and porcine parvovirus. However, due to their chemistry, PI systems are only able to target pathogens that contain nucleic acids and consequently they are ineffective against prions and transmission of variant Creutzfeldt–Jakob disease (52).

In order to demonstrate clinical efficacy, several large clinical trials using these PI systems have been conducted or are underway (22, 53) and extensive hemovigilance studies have also been undertaken. The main message is that PI treatment damages the platelets in many ways including alterations in membrane integrity, signaling pathways and in some capacity functionality of miRNAs, which results in reduced recovery and survival in healthy volunteers (54, 55). Similarly, shorter transfusion intervals have been observed in patients receiving PI-treated platelets, but these observations for the most part have not been associated with increased World Health Organization grade 2 or greater bleeding in patients receiving pathogen-reduced platelets, as hemostatic efficacy seems to be maintained (22, 26). Furthermore, some evidence points toward the fact that transfusion of PI-treated platelets does not affect mortality, the risk of clinically significant or severe bleeding, or the risk of a serious adverse event (AE) (56). However, as pointed out by Kaiser-Guignard and colleagues, the results of the published clinical studies should be interpreted with caution, and their characteristics and possible biases should be taken into account (22), such that interpretation of clinical outcome data cannot be generalized across different PI systems (22). A recent systematic review presented strong evidence that transfusion of PI-treated platelets appears to increase the risk of platelet refractoriness and the frequency of platelet transfusions (56).

The majority of contributions to investigations of PIs are *in vitro* quality studies. Multiple analyses have been conducted to monitor potential changes in the platelet quality following illumination with the three different PI systems in combination with products of different characteristics (see Table 1). These studies have typically measured common blood banking parameters, including metabolic activities, platelet activation, and platelet function to evaluate product quality, and to determine whether quality control requirements of the individual jurisdictions were met. Comparisons of different studies; however, are hampered by the fact that these measures are influenced by the type and proportion of the platelet storage medium. PI treatment of platelets in different PAS differentially alters platelet quality features (57). Recent studies with the riboflavin/UV system (MIRASOL) revealed that the quality of platelets is similar whether stored in plasma or PAS; however, transfusion of treated PCs in PAS led to fewer transfusion reactions (58). This observation is corroborated by the finding that PAS seems to have a protective effect on platelets upon illumination (59).

Based on these diverse studies, in recent years, many (individual) opinions have been published outlining the pros and cons of PI in light of safety and efficacy (20, 60–64). Ongoing discussions are guided by experiences from blood centers that have implemented PI (65–67).

## Platelet Storage Lesion (PSL): A General Overview

Many studies measuring changes to platelet *in vitro* quality indicate that PI treatment accelerates the progression of the PSL. This term describes the sum of all the deleterious changes in platelet structure and function that arise from the time the blood is withdrawn from the donor to the time the platelets are transfused to the recipient (68–73). It is mainly explained by triggering platelet activation during preparation and handling of PCs, especially the heightened metabolic activity and activation-specific changes to surface glycoproteins observed in stored platelets (74). Transient derangement of platelet metabolism can be rescued by plasma replacement, resulting in improved morphology scores, stabilized osmotic recovery, and completely restored platelet secretory responses (75).

## The Impact of PI on Platelet Functions

### PLT Activation, Degranulation, and Protein Release

As mentioned above, the main feature of PSL seems to be platelet activation, which is commonly determined by the expression of P-selectin (CD62P) on the platelet surface, as a consequence of the release of the alpha-granule content. Many studies have shown that PI increases the surface expression of CD62P (58, 76–78).

Additional features of storage-mediated platelet activation are the increased phosphatidylserine (PS) externalization (79) and changes in the protein profile of platelet surface receptors (80, 81) which are further altered upon PI treatment (82).

Among other changes, the level of cytokines and chemokines also increases in the supernatant of the storage solution during platelet storage (83–85). Although some controversy continues in the literature (86), PI treatment appears to induce platelet degranulation, hence further increasing the levels of immune factors under some treatment conditions (86–91). The altered releasate composition may affect the immunomodulatory capacity of platelets. As a consequence of this accumulation, supernatants of MIRASOL PI-treated platelets can suppress LPS (lipopolysaccharide)-induced monocyte IL-12 production (92), as well as increase LPS-induced mononuclear cell production of IL-8 (93). A recent study has demonstrated that increased supernatant levels of pro-inflammatory molecules resulting from platelet granule release are associated with reactive oxygen species generation during storage (94). This finding is corroborated by an observed increased ROS production in MIRASOL PI-treated PCs (77, 95).

A brief summary is provided in Table 2 highlighting the changes of platelet storage features by the individual PI systems.

**Table 2:** Summary of impact of pathogen inactivation (PI) treatment on platelet features compared to untreated control.

Platelet storage feature	PI system		
	
	INTERCEPT	MIRASOL	THERAFLEX
Metabolic activity	± (96); ↑ (97)	↑ (98)	↑ (99)
Platelet activation (CD62P expression)	↑ (96, 100)	↑ (98)	↑ (99)
Platelet adhesion (under flow)	± (101); ↑ (102)^a^	↓ (102); ±(103)	n.d.
Clot formation (thrombo-elastography)	↓ (104)	↑^b^, ↓^c^ (105)	↓ (99)
Responsiveness (to agonists)	↓ (102); ±↓^d^ (106)^c^	↓ (98)	± (99)
Platelet apoptosis (PS exposure)	± (107); ↑ (108)^a^	↑ (109)	↑ (99)
Platelet microparticle release	↑ (110)	↑ (111)	↑ (112)
Free mitochondria release	n.d.	↑ (95)	n.d.

*↓ = decrease; ± = similar; ↑ = increase; n.d. = not determined. The references are only examples of published studies, but are not comprehensive. Differences in some study outcomes could be due to variations in production methods used (platelet-rich plasma vs BC/PCs or apheresis PCs), composition in storage solution—plasma vs platelet additive solution (in different concentration)—and assay procedures*.

*^a^At end of storage*.

*^b^Thrombus stability*.

*^c^Aggregation*.

*^d^Agonist-dependent*.

### Development of Platelet Apoptosis

There is an ongoing debate regarding the extent to which platelet activation and programmed cell death (apoptosis) in platelets overlap at the molecular level (113). Platelets contain most of the apoptotic machinery, including pro- and anti-apoptotic Bcl-protein family members as well as caspases (114). Activation of these pathways leads to microvesiculation with expression of PS in the outer layer of the platelet membrane (115). As PS exposure is believed to contribute to the development of inflammatory or immunomodulatory processes and ultimately regulates clearance of platelet from circulation, PS exposure monitored by annexin-V binding is commonly used to measure the development of platelet apoptosis.

Pathogen inactivation treatment also results in the externalization of PS (59, 116, 117). MIRASOL PI-treated PLTs exhibit an increased expression of proapoptotic proteins Bak and Bax, but not anti-apoptotic proteins Bcl-XL (109, 116). Additionally, MIRASOL PI-triggered activation of caspase cleavage leads to proteolytic cleavage of their respective substrate proteins (116). Similar results have recently been shown in INTERCEPT PI-treated platelets (118). However, these features are not prominent until later in storage (typically 5–7 days) and may only need to be considered in the context of extended platelet storage.

### Microvesicle (MR) Release

Platelets are known to generate heterogeneous populations of cell-derived MVs (119). Platelet MVs have a bilayered phospholipid structure exposing procoagulant PS and expressing various membrane receptors, and they serve as cell-to-cell shuttles for bioactive molecules such as lipids, growth factors, microRNAs (miRNAs), and mitochondria (120). Further, the presence and quantity of MVs has been associated with the clinical severity of the atherosclerotic disease, diabetes, and cancer (6, 121). These features along with the observation that the number, function, and content of MVs in the components varies with age, gender, lipid, and hormone profiles of the blood donor (122) makes them one of the most discussed, controversial, and interesting topics in current blood banking and transfusion medicine (123). Different studies have demonstrated that all UV-based PI treatments increase the release of MVs from platelets compared to untreated controls (36, 95, 112, 124). To our knowledge, no study has directly addressed the impact of INTERCEPT on the release of MVs during PC storage; however, Kanzler et al. found a reduction of MVs in the platelet product immediate after INTERCEPT treatment (125).

### Role of Platelets in Inflammation

Although once primarily recognized for their role in hemostasis and thrombosis, platelets have been increasingly recognized as a multipurpose cell. There is growing recognition of the critical role of platelets in inflammation and immune responses. Platelets release numerous inflammatory mediators such as RANTES or CD40L, modifying leukocyte and endothelial responses to a range of different inflammatory stimuli (88). Additionally, platelets form aggregates with leukocytes and form bridges between leukocytes and endothelium, largely mediated by platelet P-selectin. Through their interactions with monocytes, neutrophils, lymphocytes, and the endothelium, platelets are, therefore, important coordinators of inflammation and both innate and adaptive immune responses. As mentioned above, studies have shown that MIRASOL-treated platelets release such mediators (92, 93) and, therefore, might modulate inflammatory responses.

### Mitochondria and Mitochondrial DNA (mtDNA) Release

Mitochondria are known as the powerhouse of cells and play a crucial role in maintaining platelet function throughout platelet storage (126). Mitochondria are released from activated platelets and upon hydrolysis of the mitochondrial membrane release mtDNA (127). MIRASOL-PI treatment also causes release of free mitochondria, mainly at the later stages of storage (95). Potentially associated with the mitochondria release, free mtDNA has been associated with AEs following platelet transfusion, and may be predictive of some types of AEs (128). mtDNA is a highly potent inflammatory trigger (128) that can be released from platelets during storage (129). Illumination of platelets with PI systems modifies mtDNA (129–131). Detection of PI-modified mtDNA using PCR assays can be used to monitor and confirm PI treatment (131). Furthermore, the relationship of mtDNA levels and AEs related to immunomodulation should also be considered; with a recent study showing an association between mtDNA and the incidence of respiratory distress posttransfusion (132).

### MicroRNA

MicroRNAs are small (~20–24 nucleotides) RNA sequences generated by ribonucleases in the nucleus (by Drosha) and cytosol (by Dicer 1) through sequential enzymatic trimming of double stranded miRNA precursors. miRNAs are thought to fine tune gene expression through degradation of their mRNA targets (133). Although platelets are anucleate, high-throughput sequencing has revealed that human platelets harbor a complex array of miRNAs, which are key regulators of mRNA translation in different cell types (134). Activated platelets can deliver mRNA regulatory Argonaute-2 miRNA complexes to endothelial cells *via* MVs leading to modulation of cell function (135).

INTERCEPT, but not MIRASOL PI treatment has been shown to affect the platelet mRNA transcriptome (27, 136). However, miRNA synthesis and function were not affected and no cross-linking of miRNA-sized endogenous platelet RNA species was observed; rather miRNA levels were reduced (136, 137). Further, the reduction in the platelet miRNA levels induced by INTERCEPT correlated with platelet activation and an impaired platelet aggregation response to ADP (136). In contrast, a recent study presented by Arnason et al. (138) demonstrated that INTERCEPT treatment did not change the quality or significantly altered the miRNA profile of PCs. These controversial results prompted further investigations and as the clinical significance of MV-associated miRNAs is unknown, and speculation of a negative effect of PI-treated platelets including long-term consequences for recipients is as yet unwarranted. This is a relatively new area of research, and additional studies are required to fully understand the impact of PI treatment on miRNA synthesis and the resulting impact on platelet quality.

### mRNA Levels and Protein Synthesis

Although anucleate, platelets have the capacity to synthesize biologically relevant proteins that are regulated *via* gene expression programs at the translational level in response to physiological stimuli (139–141). Recent studies have demonstrated that levels of specific mRNA species are reduced following MIRASOL PI treatment while others are less affected (142). Subsequent studies have revealed that this observation is mirrored in the platelet translatome, demonstrating that platelets are still capable of synthesizing proteins following PI treatment, suggesting that they may possess mechanism(s) to protect their mRNA from damage by the PI treatment (143). The clinical relevance of this finding, however, is still unknown.

### Impact of PI Treatment on Platelet Lipidomics

Although the application of lipidomics to platelet biology is still in its infancy, seminal studies have shaped our knowledge of how lipids regulate key aspects of platelet biology, including aggregation, shape change, coagulation, and degranulation, as well as how lipids generated by platelets influence other cells, such as leukocytes and the vascular wall, and thus how they regulate hemostasis, vascular integrity, and inflammation, as well as contribute to pathologies, including arterial/deep vein thrombosis, and atherosclerosis (144). Mapping the human platelet lipidome revealed cytosolic phospholipase A2 as a regulator of mitochondrial bioenergetics during activation (145). A recent study has demonstrated that psoralen and UV light increased the order of lipid phases by covalent modification of phospholipids, thereby inhibiting membrane recruitment of effector kinases such as BTK and Akt and consequently affecting GPVI- and PAR1-mediated signal transduction (99).

## Further Investigations toward Understanding the Molecular Mechanisms of PI-Induced Platelet Alteration: From Proteomics to Signaling

A variety of untargeted proteomic approaches have been used to assess the impact of PI systems on platelets (146–148). The effect of the PI treatment on the proteome appears to be different according to the particular technology. A comparative analysis of proteomic data revealed that MIRASOL seems to impact proteins involved mainly in platelet adhesion and shape change while INTERCEPT affects proteins of intracellular platelet activation pathways and THERAFLEX influences proteins linked to platelet shape change and aggregation (149). These conclusions are based on a relatively small number of studies and further analyses are required for verification.

**Figure 1:**
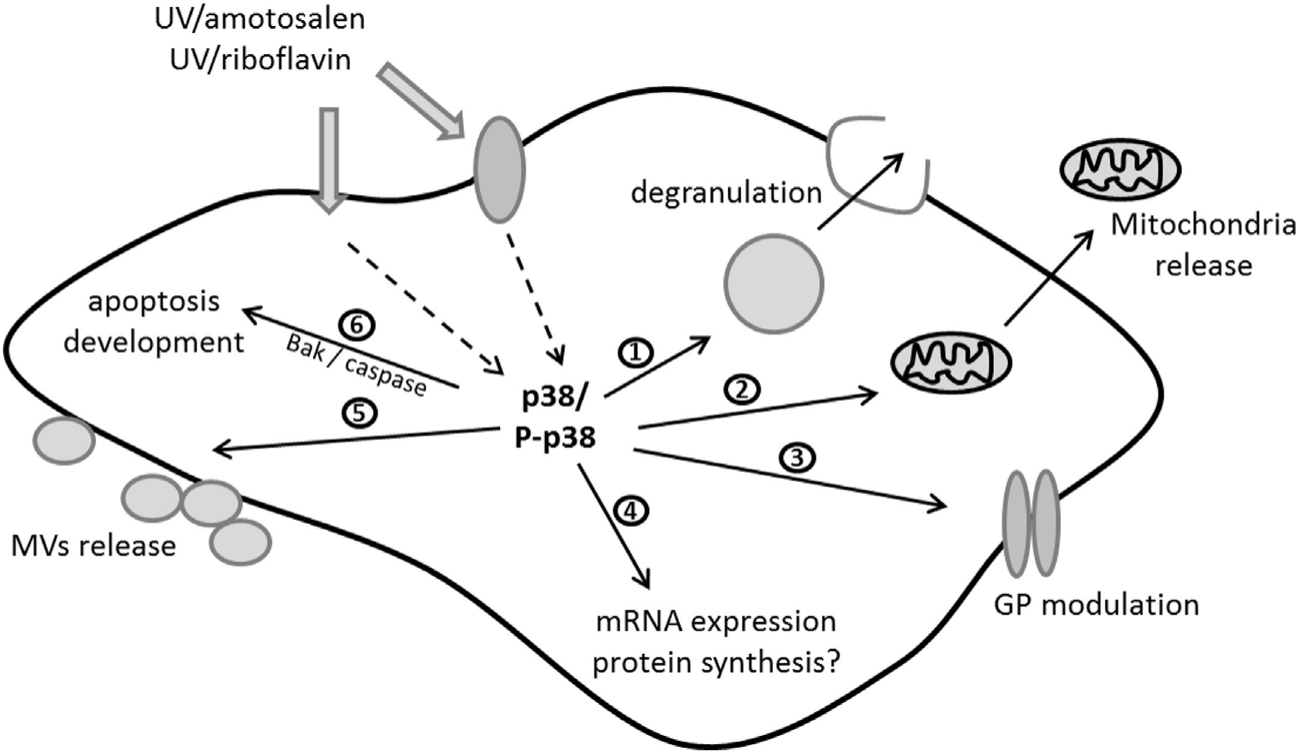
Current molecular model of signaling triggered by ultraviolet (UV)/riboflavin (MIRASOL) and UV/amotosalen (INTERCEPT) in platelets: UV can penetrate either directly or *via* surface/receptor proteins to activate p38MAPK kinase as one of the central players in the signaling cascade. Thus far it has been shown that p38 activation/phosphorylation (P-p38) is involved in regulating (1) degranulation, (2) release of free mitochondria, (3) the modulation of glycoproteins (GPs), (4) the expression levels of mRNAs and potentially protein synthesis, (5) microvesicle (MVs) release, and (6) the development of apoptosis *via* proapoptotic protein expression and caspase activation. This figure was modified from Ref. (150).

A more targeted approach using a phospho-kinase antibody-based array demonstrated that a variety of kinases were activated by MIRASOL PI treatment (150). p38MAPK plays a central role in MIRASOL PI-mediated signaling by regulating a variety of platelet features, such as apoptosis (109), mitochondrial function, and release of free and MV-encapsulated mitochondria (95). The INTERCEPT system also triggers p38MAPK activation in platelets, and the phosphorylation of the p38MAPK substrate Tace is directly linked to GPIb cleavage possibly explaining the reduced adhesion of those platelets under flow conditions (118). The role of p38MAPK in mediating PI-triggered signaling linked to features of PSL is supported by studies demonstrating a regulatory role of p38MAPK in regulating PSL development (151) and platelet *in vivo* recovery and survival in mouse models (152). This body of work suggests that similar signaling pathways are activated by both of these PI systems as modeled in Figure 1. Although only a few studies to date have investigated the signaling aspect in platelets, it could be hypothesized that p38MAPK activation in response to the stress associated with the PI treatment may have a regulatory role in platelet life span (153) as inhibition of this protein leads to decreased apoptosis (109, 118).

## Conclusion and Future Directions

Although there are numerous studies in the literature assessing the impact of UV-based PI systems on platelet *in vitro* and *in vivo* function, only a few conclusions can be drawn. All technologies seem to accelerate the development of some form of the PSL but this likely results through different modes of action; therefore, it is likely that many divergent, as well as overlapping molecular mechanisms are triggered. Most of the functional studies conducted to decipher the role of signaling pathways in PI-treated platelets have been carried out using the INTERCEPT and MIRASOL system and thus the effects of the THERAFLEX system remain relatively unknown. However, it is clear that PI-treated platelets are different to untreated platelets, and the differences may go some way toward explaining some of the clinical observations following transfusion of PI-treated platelets. Proteomic analyses and in future other -omics approaches such as metabolomics (154) will likely shed more light into the specific effects of PI treatment. Additional targeted approaches will guide the formulation of signaling models, which may ultimately identify pathways known to impact platelet function upon illumination, and provide potential (protein) markers to assist with the fine-tuning of these technologies. We need to keep in mind, however, that the PI treatment does not only affect platelets *per se*, these procedures trigger the release of MVs, proteins, and nucleic acids in to the storage medium which also gets transfused. Whether any of these components will have deleterious effects on the recipients remains to be determined even though the initial clinical studies do not show significant clinical effects from PI treatment of PCs.

## Author Contributions

All authors contributed to this manuscript and approved the final version for submission.

## Conflict of Interest Statement

The authors declare that the research was conducted in the absence of any commercial or financial relationships that could be construed as a potential conflict of interest.
